# Neuromechanical coupling during mechanical ventilation: reverse-triggering vs others forms of asynchrony

**DOI:** 10.1186/2197-425X-3-S1-A997

**Published:** 2015-10-01

**Authors:** JA Benitez Lozano, F Ruiz Ferron, P Carmona Sanchez, JM Serrano Simon

**Affiliations:** Hospital Quiron, Malaga, Spain; Complejo Hospitalario de Jaen, Intensive Care Unit, Jaen, Spain; Hospital Universitario Reina Sofia, Intensive Care Unit, Cordoba, Spain

## Introduction

The term “respiratory entrainment” refers to of coupling patterns between the rhythm neuronal and mechanical of ventilator to achieve satisfactory synchrony. Respiratory effort stimulated by the mechanical inspiratory flow or reverse trigger (RT), has been recently demonstrated [1]. This asynchrony is difficult to showed without additional signals as esophageal pressure or electrodiaphragmatic signal.

## Objectives

To use a method to differenciate this asynchrony by the standard signals of the ventilator, using an occlusion maneuver and to show the characteristics of this RT.

## Methods

Over 4-month from December-2014 we studied a group of consecutive ventilated patients because they showed too high respiratory drive, beside they were sedated and assist-volume control mechanical ventilation was changed trying improve ventilator interaction. Signals of flow, airway and pleural or esophageal pressure were registered during 3min for ulterior analysis. Passive respiratory mechanics were measured by multiple linear regression. Entrainment pattern we evaluated by measure of mechanical and neural respiratory times (Ttotm, Ttotn), and repetitive phase of ventilatoy cycles. On apparent reverse triggering we carried out an occlusion maneuver. Gasometric data was recording. We classified entrainment ratio. Data were analyzed by descriptive statistical and are expressed as mean ± SD, medians [IRQ] and coefficient of variation (CV). Comparison of ventilator and neural cycle, and phase angle of apparent reverse triggering vs total asynchronic identified by occlusion maneuvers were performed with test Mann-Whitney.

## Results

5 of 12 patients were identified for study, 4 patients postoperative lung transplant and 1 with ARDS. Age 50yr (41,25-56,25). The patients with RT (N3) showed minimal variability in neuromechanical coupling pattern and had no respiratory activity after occlusion maneuver.The periods of entrainment varying from 40 to 90% of recording time. The 1:1 pattern was dominant. For all data: Respiratory system elastance and resistances 35 ± 7,9cmH2O/L and 16,86 ± 6,6cmH2O/L/s. Mechanical and neural respiratory time 3s (2,88-3,02) and 3,1s (2,98-3,44); phase angle 61,94º(49,79-99,14). Tidal volume 0,52L ± 0,02. RR 21,20 ± 1,64/min. PaCO2 49,8 ± 8,04mmHg. CV for reverse triggering vs asynchrony: Ttotm 14% vs 14,86%; Ttotn 7% vs 16,45%; phase 17,25% vs 26,48% (p < 0,001).

## Conclusions

This method is useful to demonstrate RT vs overall asynchrony. RT is characterized by: 1) The less variability between mechanical and neural respiratory cycle involves a fixed neuromechanical coupling. 2) Absence of spontaneous breathing activity, manifested after occlusion maneuver.

**Figure 1:**
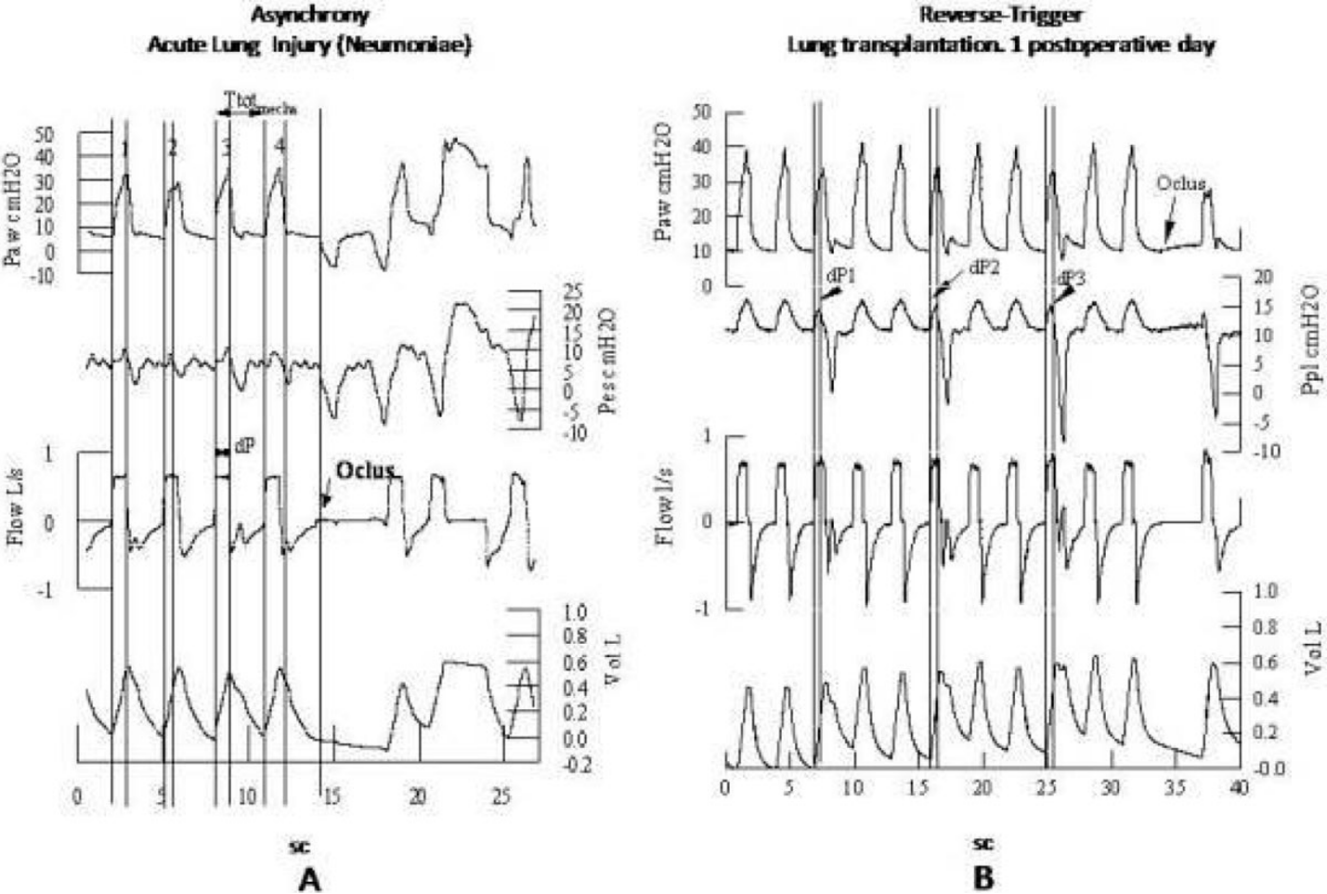
**Traces of flow, Paw, Pes o Pleural Pressure during entrainment epochs**. A, case of asynchrony 1:1 vs B, Reverse triggering 1:3, identified by neuromechanical coupling pattern and occlusion maneuver. Note high variability of dP in case A.
